# FDT Perimetry for Glaucoma Detection in Comprehensive Health Checkup Service

**DOI:** 10.1155/2020/4687398

**Published:** 2020-03-29

**Authors:** Ryo Terauchi, Takashi Wada, Shumpei Ogawa, Masanobu Kaji, Tomohiro Kato, Masayuki Tatemichi, Tadashi Nakano

**Affiliations:** ^1^Department of Ophthalmology, The Jikei University School of Medicine, Tokyo 105-8461, Japan; ^2^Health Science, The Jikei University School of Medicine, Tokyo 105-8461, Japan; ^3^Harumi Toriton Clinic, The Jikei University School of Medicine, Tokyo 104-0053, Japan; ^4^Center for Preventive Medicine, The Jikei University School of Medicine, Tokyo 105-8461, Japan; ^5^Department of Preventive Medicine, Tokai University School of Medicine, Isehara 259-1193, Japan

## Abstract

We aimed to investigate the efficacy of frequency doubling technology (FDT) perimetry for glaucoma detection in comprehensive screening examinations. We performed a retrospective analysis of prospectively collected data of participants who underwent a comprehensive health checkup service. Participants with glaucoma were excluded. In the first year, 2024 participants (46.8 ± 9.4 years) who underwent FDT perimetry and fundus photography were classified as the FDT group, whereas 3052 participants (42.2 ± 8.2 years) who underwent only fundus photography were classified as the non-FDT control group. Participants with abnormal findings on FDT perimetry and/or fundus photography were recommended to undergo further complete examination. All participants reported whether they had been newly diagnosed with glaucoma within 2 years of the first visit. In the FDT group, 23 (1.14%) participants were newly diagnosed with glaucoma. Among them, 20 (87.0%) had abnormal FDT perimetry findings and 12 (52.2%) had abnormal findings on fundus photography. The positive-predictive value (PPV) of FDT perimetry was 16.5% (20/121) and that of fundus photography was 13.3% (12/90). In participants with abnormal findings on both tests, the PPV was 26.2%. In the non-FDT group, 15 (0.49%) participants were newly diagnosed with glaucoma. Among them, 9 (60.0%) had abnormal findings on fundus photography. The PPV of fundus photography was 10.8% (9/83). The glaucoma detection rate, analyzed using age adjustment, was significantly higher in the FDT group than that in the non-FDT group (0.97% versus 0.47%, *P*=0.041). FDT perimetry, even if performed by nonspecialized physicians, could improve glaucoma detection when used in addition to fundus photography. This study was registered with UMIN000037951.

## 1. Introduction

Glaucoma is a group of eye diseases that damage the optic nerve, resulting in characteristic visual field defects. It is estimated that the total number of glaucoma cases worldwide will rise to 79.6 million in 2020 and 111.8 million in 2040 [1, 2]. Glaucoma is the second leading cause of blindness worldwide, after cataract [3].

Normal-tension glaucoma (NTG) accounts for 72.4% of all glaucoma cases in the Japanese population [4] and is the main target of glaucoma screening. However, NTG is often detected in the advanced stage with severe visual field defects because almost all patients have no subjective symptoms, particularly in the early and less advanced stages of the disease. The Tajimi study, a population-based prevalence survey of glaucoma in Japan, estimated that 95.5% of the NTG cases were previously undiagnosed [4]. Although glaucomatous visual field defects are progressive and irreversible, early detection can enable prevention of these defects. Therefore, early detection of glaucomatous changes and early treatment intervention are very important.

Frequency doubling technology (FDT) perimetry has been used as one of glaucoma screening tools. Previous studies have already shown its effectiveness for detecting glaucomatous visual field defects [5–7]. Magnocellular cells, which are large-diameter retinal ganglion cells transmitting visual information, are damaged in early glaucoma [8–10]. FDT perimetry can selectively detect the loss of function of these cells [10, 11].

Some types of health screening examinations are required by law in Japan. Unfortunately, these do not include an ophthalmic examination, except for a visual acuity test. However, there is also comprehensive health checkup service intended to provide a complete evaluation of various organs, including the eyes. Examinees undergo medical history taking, physical examination, blood and urine sampling, and radiological imaging. In case of abnormal test results, further detailed evaluation in specialized medical institutions is recommended. The cost for these examinations is sometimes covered by the examinees' insurance, particularly among those insured by the Society-Managed Health Insurance.

The standard ophthalmic examinations in comprehensive health checkup are the visual acuity test and fundus photography. However, NTG does not affect the visual acuity in the early and less advanced stages of the disease. Hence, we depend only on fundus photography for the detection of NTG. Additionally, in many cases, physicians who evaluate the fundus images are not specialized in ophthalmology. Fundus image evaluation requires skills, as glaucomatous changes are difficult to detect, even for trained ophthalmologists. Given the above conditions, it is considered that comprehensive health checkup would be insufficient for glaucoma screening.

In this study, we introduced FDT perimetry as a glaucoma screening tool and investigated the glaucoma detection rates one year after the screening tests. The Harumi Triton Clinic, Tokyo, Japan, provides different types of comprehensive health checkup service depending on the price. Regarding the eye examinations, there are two different types: type A, which includes the visual acuity test and fundus photography, and type B, which includes FDT perimetry in addition to the previous tests. We compared the glaucoma detection rates of these two types of examinations. To the best of our knowledge, there has been no study on the glaucoma detection rates using FDT perimetry that directly compared these two types of examinations in the same facility during the same period.

## 2. Materials and Methods

### 2.1. Study Participants

We performed a retrospective analysis of prospectively collected data of participants who underwent a comprehensive health checkup service at the Harumi Triton Clinic between January 2002 and December 2015. Participants who had not been diagnosed with glaucoma and without a history of retinal disease at the first visit and revisited the clinic within 2 years were included in the analysis. In the first year, all participants underwent a visual acuity test with Landolt C rings and fundus photography with CR6-45NM Nonmydriatic Retinal Camera (Canon Inc., Tokyo, Japan). According to the selected medical examination type, some participants additionally underwent FDT perimetry using the Humphrey FDT perimeter (Carl Zeiss Meditec, Dublin, CA). These were classified as the FDT group and the remaining participants were classified as the non-FDT group. Almost all participants in the Harumi Triton Clinic were employees who were requested to undergo a periodical health checkup by their employers. The medical examination type for each participant was determined based on the contract between their employer and Harumi Triton Clinic. That is, the examination type selection was not related to the participants' individual intentions.

At the first visit, all participants signed a consent form titled “Use of data obtained from medical examination for medical research.” Only participants who provided consent were included. This study was approved by the Institutional Review Board of the Jikei University School of Medicine (approval number: 30-309 [9330]). Prior written informed consent was obtained from all participants. The study is registered with UMIN000037951.

### 2.2. FDT Perimetry

The participants of the FDT group underwent FDT perimetry with habitual correction in place. The test was orally explained to each participant, and a preview of the target stimuli was shown at the beginning. Participants were instructed to stare at the black dot in the center of the screen during the entire test and press the response button once upon seeing the flickering black and white vertical bars in the screen.

**Figure 1:**
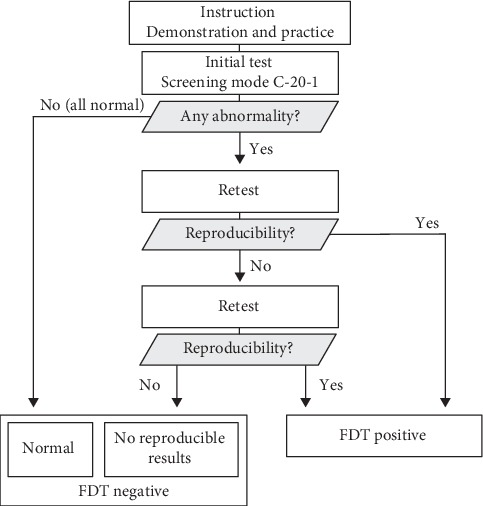
FDT perimetry screening protocol.

In this study, the screening C-20-1 program was used, which tested 17 visual field locations. After the test, each test location was classified into one of four grades: “within normal limits,” “mild relative loss,” “moderate relative loss,” and “severe loss.” We considered “within normal limits” as normal, and the other three grades as abnormal.  Figure 1 shows our FDT perimetry screening protocol. When the initial FDT perimetry test showed ≥1 spots of abnormality, we defined it as abnormal and immediately performed a retest. The test was defined as positive when the spots of abnormality in the retest were the same as or in contact with the abnormal ones in the first test. When there was no reproducibility between the first and the second test results, an additional test was performed after a 5-min break. The third test result was compared with the first and second results. If the third test reproduced the first and/or second test, we classified the FDT test as positive. When there was no reproducibility among three tests, we classified the FDT test as negative.

### 2.3. Ocular Fundus Photography

All participants underwent ocular fundus photography with nondilated pupils. The general physicians detected the cup-to-disc ratio (CDR), retinal nerve fiber layer defect (RNFLD), notching, and disc hemorrhage (DH). We considered a CDR of 0.7 or greater as abnormal. The participant was suspected of having glaucoma if any one of four findings was found. Although physicians had a thorough knowledge about abnormal findings of fundus photography, each physician had a different level of experience. Two physicians graded one fundus image. Seven physicians were involved with fundus grading during the study period.

### 2.4. Data Collection

In case of a positive FDT perimetry test and/or abnormal fundus photography findings, except in participants under treatment, an ophthalmologist consultation was recommended for a complete evaluation and treatment as necessary. We determined the number of participants with newly diagnosed glaucoma based on their self-report at the second visit. Namely, on their subsequent visit, all participants completed medical questionnaires wherein they reported whether they had been newly diagnosed with glaucoma. We also obtained information from the participants' referral documents from the specialized medical institution where they had undergone a complete ophthalmological examination and treatment.

### 2.5. Statistical Analysis

The data were expressed as means ± standard deviation. The clinical findings were statistically evaluated using R, version 3.6.1. (R Foundation; http://r-project.org). The Mann–Whitney *U* test was used to compare the differences between the two groups. The chi-square test was used to determine the differences between percentages. The glaucoma detection rates after age adjustment between the FDT and non-FDT groups were compared using a general linear model. A *P* value <0.05 was considered to indicate statistical significance.

## 3. Results

### 3.1. Characteristics of the Study Participants

A total of 5153 participants revisited our clinic within 2 years of the first visit. Of these, 77 (1.49%) patients with glaucoma were excluded. Finally, this study enrolled 5076 participants (3489 men and 1587 women). The participants were assigned to the FDT group (2024 participants) or the non-FDT group (3052 participants) as described in Section 2.1. The mean age of the FDT group was significantly higher than that of the non-FDT group (46.8 ± 9.4 years and 42.2 ± 8.2 years, respectively). The proportion of males in the FDT group was significantly lower than that in the non-FDT group (64.3% and 71.7%, respectively). There was no significant difference in the visual acuity between the two groups (Table 1).

### 3.2. Ophthalmologic Examination Results and Glaucoma Detection Rates

In the FDT group, the FDT perimetry test was abnormal in 121 (6.0%) participants. The proportion of abnormal fundus photography results in the FDT group was significantly higher than that in the non-FDT group (4.4% (90/2024) versus 2.7% (83/3052), respectively). Twenty-three (1.14%) participants in the FDT group and 15 (0.49%) in the non-FDT group were newly diagnosed with glaucoma. There was statistically significant difference in the glaucoma detection rate between FDT group and non-FDT group using the general linear model for age adjustment (Table 2).

### 3.3. FDT Group

The FDT perimetry and ocular fundus photography results in the FDT group are shown in Table 3. The positive-predictive value (PPV) of FDT perimetry was 16.5% (20/121). Among the 23 participants diagnosed with glaucoma, 20 (87.0%) had a positive FDT perimetry test. The PPV of fundus photography was 13.3% (12/90). Eleven (47.8%) of the 23 participants diagnosed with glaucoma had abnormal fundus photography findings. Participants with abnormal results on both tests had a higher probability (26.2%) of being diagnosed with glaucoma than those with abnormal results only on the FDT perimetry test (11.4%) or only on fundus photography (2.1%). The glaucoma detection rate was 0.11% in participants with normal results on both tests.

### 3.4. Non-FDT Group

The ocular fundus photography results in the non-FDT group are shown in Table 4. The PPV of fundus photography was 10.8% (9/83). Nine (60.0%) of the 15 participants diagnosed with glaucoma had abnormal fundus photography findings.

## 4. Discussion

In this study, we demonstrated that FDT perimetry, even if performed by general physicians not specialized in ophthalmology, could improve glaucoma detection in comprehensive health checkup service, in combination with fundus photography.

To evaluate the effectiveness of FDT perimetry as a glaucoma screening tool, we divided the participants into two groups, FDT group and non-FDT control group, and we investigated the number of participants with newly diagnosed glaucoma. To the best of our knowledge, there has been no study that directly compared the groups in the same facility during the same period. This excluded the regional socioeconomic bias in the between-group comparison. Further, the participants were mostly employees who were assigned to either of the two groups according to the contract between their employer and the Harumi Triton Clinic. This excluded self-selection bias in the between-group comparison.

The Humphrey field analyzer (HFA) is the gold standard for the diagnosis of glaucoma. However, cost-effectiveness and time constraints need to be considered in mass glaucoma screening settings, in which a large number of individuals are examined every day. The main purpose of mass screening for glaucoma is not the diagnosis of glaucoma but referring those suspected of having glaucoma to specialized hospitals. Glaucoma suspects can visit an ophthalmologist and undergo complete examination including HFA without time and cost restrictions. We intentionally selected FDT and not HFA for this study after considering testing time, cost, size, and the need for a dark room.

Many previous studies have reported FDT perimetry to be useful as a glaucoma screening tool [5–7, 10, 12–14]. Iwasaki and Sugita [5] demonstrated that FDT perimetry could detect 83.3% and 100% of patients with glaucoma in the early and more advanced stages, respectively. In addition, FDT perimetry has some advantages, such as a short testing time, low cost, and compact size. However, some previous studies have concluded that FDT perimetry had some limitations as a screening tool due to the low sensitivity and specificity [15–17]. We concluded that the lack of a gold-standard FDT perimetry protocol for glaucoma screening might have caused such conflicting results. These previous studies had many differences in the FDT perimetry protocol, including differences in the study participants, screening mode, criteria for abnormalities, number of tests performed, and the method of definitive diagnosis of glaucoma. Therefore, it would be very important to provide an appropriate test protocol for satisfactory performance of FDT perimetry in glaucoma screening.

The combined PPV of FDT and fundus photography was 26.2%. Assuming that the prevalence of glaucoma in our study population ranged from 2% to 3% and the sensitivity and specificity of screening tests were 90% and 90%, respectively, the PPV value ranged from 18% to 27%. This study demonstrated the potential feasibility of glaucoma screening using FDT perimetry in real-world primary care settings. However, efforts should be made to improve the efficiency of the screening method. Iwasaki and Sugita [5] achieved satisfactory detection rate of glaucoma using FDT perimetry by focusing on the “glaucoma area,” which consists of four spots in the center on the nasal side. Considering the characteristic patterns of glaucomatous visual field loss in FDT would be useful. The threshold test could also improve the screening performance of FDT. We intentionally selected the screening mode and not the threshold mode after considering the testing time. The threshold mode requires more testing time than that with the screening mode, which does not satisfy the requirement for mass screening for glaucoma. Moreover, intermediate steps including telemedicine between initial screening and visiting an ophthalmologist have the potential to effectively improve the PPV.

In our study, 83 participants (2.7%) in the non-FDT group and 169 participants (8.3%) in the FDT group were recommended to undergo further ophthalmologic examination. The high rate of abnormal results in the FDT group might have contributed to a higher glaucoma detection rate. However, we could not verify this hypothesis because we did not follow-up whether each participant with an abnormal result visited an ophthalmologist or not.

The rate of abnormal fundus photography findings was significantly higher in the FDT group than in the non-FDT group. This result remained unchanged after age adjustment. We believe that the reason for this is that the general physicians had a tendency to evaluate the fundus photography results as abnormal in participants with a positive FDT perimetry test. They assessed the fundus images while referring to the FDT perimetry results. In the aftermath, the PPV of fundus photography in the FDT group was higher than that in the non-FDT group. This finding indicates that it is difficult to objectively evaluate fundus images, particularly for general physicians not specialized in ophthalmology.

When comparing the characteristics of the two groups, there were significant differences in the age and gender. Aging is one of the major risk factors for glaucoma [18–20]. Therefore, we performed age adjustment to eliminate the effect of aging in the comparative analysis of the two groups. Meanwhile, there is no clear consensus on gender predilection for primary open-angle glaucoma, including NTG [21].

The standard ophthalmologic examinations in the comprehensive health checkup in Japan are the visual acuity test, intraocular pressure (IOP) measurement, and fundus photography. However, we did not perform IOP measurement. When comprehensive health checkup service was launched in 1954, only the visual acuity test and fundus photography were employed. IOP measurement was additionally introduced mainly as a glaucoma screening tool in 1975. Yet, the Tajimi Study in 2004 demonstrated that NTG accounted for 72.4% of all glaucoma cases [4]. This finding indicated that IOP measurement could not detect the majority of glaucoma cases. Another previous study demonstrated that IOP measurement was not cost-effective for glaucoma screening [22]. Recently, IOP measurement devices such as iCare (Tiolat Oy, Helsinki, Finland) have been used in the clinical setting. Devices such as iCare are inexpensive, portable, easy to operate, relatively accurate, and do not require local anesthesia, which is compatible with glaucoma mass screening. Although we did not measure the IOP in this study, future studies should assess whether iCare can improve the efficacy of glaucoma screening.

As with iCare, optical coherent tomography (OCT) is also expected to be a new glaucoma screening tool in primary care setting. OCT, which can detect glaucomatous structural changes in the optic disc and retina [23], has already been adopted widely in clinical settings. Modern OCT technology is easy to use with nondilated pupils and is becoming increasingly cost-effective. In future studies, we should reconsider the most effective combination of multiple ophthalmic examinations, including new devices such as OCT and iCare.

A limitation of this study was that the true number of patients with glaucoma in the following year was unclear. Although participants with a positive FDT perimetry test and/or glaucomatous findings on fundus photography were recommended to see an ophthalmologist and undergo a complete examination, some participants probably did not undergo additional examination by the following year. Participants potentially undiagnosed with glaucoma were not counted as newly diagnosed with glaucoma. For this reason, the PPVs of FDT perimetry and fundus photography were low values. If more participants with suspected glaucoma could be referred to a specialized medical institution, the PPV would further improve. This is our challenge for the future. Further, newly diagnosed glaucoma was based on self-reported data obtained from medical questionnaires at the second visit, which could have an effect on the PPVs. It should be also noted that the age distribution of our participants did not match that of Japan, since this study mainly targeted middle-aged workers.

## 5. Conclusions

FDT perimetry is a useful glaucoma screening tool and may improve the effectiveness of glaucoma detection when used in combination with fundus photography. It is important that an appropriate FDT perimetry protocol is developed to satisfactorily detect early glaucoma during screening. In future studies, we should determine the most effective combination of multiple ophthalmic examinations, including new devices such as OCT and iCare.

## Figures and Tables

**Table 1:** Characteristics of the study participants.

Variable		FDT	Non-FDT	*P* value
		*n* = 2024	*n* = 3052	
Age	Mean (SD)	46.8 (9.4)	42.2 (8.2)	<0.001
Gender	Male (%)	64.3	71.7	<0.001
LogMAR visual acuity, OD	Mean (SD)	0.052 (0.23)	0.054 (0.24)	NS
LogMAR visual acuity, OS	Mean (SD)	0.053 (0.23)	0.052 (0.24)	NS

LogMAR = logarithm of the minimum angle of resolution, SD = standard deviation, OD = right eye, OS = left eye, FDT = frequency doubling technology, and NS = not significant.

**Table 2:** Results of the ophthalmologic examinations and glaucoma detection rate.

Result type	FDT	Non-FDT	*P* value
	*n* = 2024	*n* = 3052	
FDT			
Abnormal	121 (6.0)	—	—
Normal	1903 (94.0)	—	
Fundus photography			
Abnormal	90 (4.4)	83 (2.7)	0.045
Normal	1934 (95.6)	2969 (97.3)	
Newly diagnosed glaucoma	23 (1.14)	15 (0.49)	0.034
Age-adjusted detection rate, %	0.97	0.47	0.041

FDT = frequency doubling technology.

**Table 3:** Results of FDT perimetry and fundus photography in the FDT group.

	FDT	Fundus photography	n	Newly diagnosed glaucoma
Both tests abnormal	Abnormal	Abnormal	42	11 (26.2%)
Abnormal FDT perimetry	Abnormal	Normal	79	9 (11.4%)
Abnormal fundus photography	Normal	Abnormal	48	1 (2.1%)
Both tests normal	Normal	Normal	1855	2 (0.11%)
Total	—	—	2024	23 (1.14%)

**Table 4:** Results of fundus photography in the non-FDT group.

	*n*	Newly diagnosed glaucoma
Abnormal	83	9 (10.8%)
Normal	2969	6 (0.20%)
Total	3052	15 (0.49%)

## Data Availability

The datasets used to support the findings of this study are available from the corresponding author on reasonable request.
